# The First-year Integration Test: a validation study

**DOI:** 10.3389/fpsyg.2023.1101234

**Published:** 2023-08-10

**Authors:** Jonas Willems, Veerle Vanoverberghe, Liesje Coertjens, Vincent Donche

**Affiliations:** ^1^Department of Training and Education Sciences, University of Antwerp, Antwerp, Belgium; ^2^Artevelde University College Ghent, Ghent, East Flanders, Belgium; ^3^Psychological Sciences Research Institute, Université Catholique de Louvain, Ottignies-Louvain-la-Neuve, Walloon Brabant, Belgium

**Keywords:** transition to higher education, first-year academic experience, first-year social experience, professional higher education, validating a measure

## Abstract

To date, there has been little agreement on what important facets should be incorporated in quantitative measures of the academic and social first-year experience. Moreover, such scales are typically developed in academic higher education (HE) contexts (i.e., universities providing more theoretical and scientific education), and are disconnected from the experiences of students in professional HE contexts (i.e., university colleges, offering more vocational education that prepares students for practicing a specific occupation, such as social work). The First-year Integration Test (FIT) is a newly developed instrument that aims to map out students’ perceptions of how they are dealing with their academic and social transition in the first semester of HE, and was based on thorough qualitative research in professional HE. In the present study, the psychometric value of FIT is explored. Results show that FIT has good construct validity, and all scales are internally consistent. Also, evidence was found for the convergent, discriminant and criterion-related validity of the instrument. We argue that FIT holds potential as a tool for generating student feedback, as a monitoring tool within an institution’s quality assurance system, and as a valuable research instrument.

## 1. Introduction

A student’s transition into the First-Year of Higher Education (FYHE) is accompanied with a complex set of new academic and social requirements that need to be successfully navigated (Credé and Niehorster, 2012). First-year students indeed are confronted with the necessity to, for instance, manage a high workload, develop new academic skills, become a more self-regulated learner or build new peer relationships (e.g., Trautwein and Bosse, 2017; De Clercq et al., 2018). The first-year student transition experience has been subject to considerable research, as it is generally acknowledged that effectively adapting to the social and academic spheres of the new HE learning environment, positively affects important student success outcomes, such as academic achievement and mental well-being (e.g., Bowman, 2010; Richardson et al., 2012; Tinto, 2012).

Over the years, a multitude of measures has been developed to assess first-year students’ academic and social transition experiences, oftentimes building on the multi-faceted “umbrella” constructs *academic* and *social integration* and *adjustment* (e.g., Tinto, 1975, 2007; Baker and Siryk, 1984). In general, however, there is substantial theoretical ambiguity surrounding these constructs, and scholars do not seem to agree on which important sub-facets of the transition process should be incorporated in such quantitative measures (e.g., Davidson and Wilson, 2013). Furthermore, to the best of our knowledge, integration and adjustment scales are typically developed in academic HE contexts (i.e., universities offering theoretical and scientific education), and are rather disconnected from the experiences of students in more professional HE programs, offering more vocational education that prepares students for a specific occupation, such as social work. Worldwide, however, a significant number of students participate in professional HE (OECD, 2009). In Flanders (Dutch speaking part of Belgium), for instance, 48.8% of students begin their HE careers enrolled in a professional bachelor program (Flemish Government, 2021).

In light of these paucities, this study builds on a recently developed theoretical framework, which was based on the findings of substantive qualitative research, and which delineates a comprehensive view on the most important (critical) experiences at play during the academic and social transition process of students in professional HE (Willems et al., 2021a,b). The goal of the present study is to provide a quantitative translation of the constructs that are described in this theoretical framework, resulting in a valid and reliable questionnaire that offers a picture on how students are dealing with their social and academic transition at a certain point in the first semester. Such a questionnaire might, then, be used (1) to provide first-year students with personal feedback on their transition process, as a vehicle for student reflection, (2) as a monitoring tool, that can be part of the institution’s quality assurance system, or (3) as a research instrument (Parpala and Lindblom-Ylänne, 2012; Kyndt et al., 2017).

In what follows, we firstly characterize the HE system of Flanders (the Dutch speaking part of Belgium), where this study has taken place, after which we describe how academic and social experiences in FYHE can impact student success in HE. Finally, we critically discuss how first-year students’ adjustment and integration is currently often measured in only academic HE contexts.

## 2. Research context: the Flemish HE system

In Flanders, HE is offered by two types of HE institutions. Firstly, *university colleges* organize professional bachelor programs which offer a direct access to the labor market and coincide to the Bologna first cycle programs [one cycle of 3 years; The Bologna Declaration (1999)]. Secondly, *universities* organize academic bachelor programs which prepare students for a master program and correspond to the Bologna two-cycle programs (bachelor and master, encompassing a total of 4 or 5 years). Such a dual HE system, wherein academic and professional HE is organized within separate, specialized institutions, can also be found in many other countries, such as Germany, the Netherlands, Finland, Denmark and Portugal (Camilleri et al., 2014).

Academic and professional HE programs differ from each other with regard to their aims and expectations of students, and oftentimes have a disparate curricular organization. Academic programs typically provide theoretical and scientific education, encompassing subject matter that is more abstract and often less practical than in professional education. Also, in academic programs the teaching speed is generally higher, and more self-regulated learning is expected from students (van Rooij et al., 2018a). In professional HE programs, on the other hand, students are taught more tailored knowledge, skills and competencies specific to a particular occupation (e.g., nursing or social work). Students in these vocationally oriented programs typically are confronted with more student-centered and collaborative learning settings, such as long-term internships, and extensive small-group practice lessons (Camilleri et al., 2014).

## 3. The impact of academic and social transition experiences on students’ success

The importance of students’ academic and social transition experiences during their FYHE, and their subsequent impact on important student success outcomes, is widely acknowledged in scholarly literature (Krause and Coates, 2008; Jansen and van der Meer, 2012; Tinto, 2012; Gale and Parker, 2014). There are multiple conceptualisations of student success in HE. A first, more traditional, perspective is concerned with the question if and why first-year students are successful in terms of their academic achievement. This product oriented perspective, focuses on hard, objective outcome variables, such as grade point average (GPA; the average score across all examinations after a certain period) (e.g., Robbins et al., 2004; Richardson et al., 2012). A second, more recent, perspective focuses on more soft, subjective student variables (e.g., Kuh et al., 2011; Credé and Niehorster, 2012). This latter strand of research acknowledges that subjective variables, such as students’ mental well-being, are fundamentally beneficial to students (Stupnisky et al., 2013), and, thus, are essential outcome variables in their own right (van der Zanden et al., 2018). In this context and in line with Willems (2022), we conceptualize student success as encompassing both academic achievement and mental well-being of students. Recognizing the multidimensional nature of mental well-being, we adopt a comprehensive definition, which embraces two widely accepted perspectives: (1) an individual’s subjective experience of happiness and life satisfaction, known as the hedonic perspective; and (2) an individual’s positive psychological functioning, capacity to establish and maintain meaningful relationships, and pursuit of self-realization, referred to as the eudaimonic perspective (Ryan and Deci, 2001; Tennant et al., 2007).

The academic and social transition experiences encountered by students can exert influences on their academic achievement as well as their mental well-being. A substantial body of previous research has shown small to moderate correlations between academic experience scales and students’ GPA (e.g., Robbins et al., 2004; Richardson et al., 2012). The relationship between the social experience scales and academic achievement is less investigated and seems to be less straightforward. Although some studies have found connections between social experience scales and academic achievement (e.g., Robbins et al., 2004; Richardson et al., 2012), it has also been observed that various factors, such as student characteristics or the type of extracurricular activity, influence whether social experience scales have a positive or negative impact on academic achievement (e.g., Pike, 2003; Baker, 2008). Consequently, researchers have emphasized the need for further investigation into this relationship (De Clercq et al., 2018). Conversely, it is anticipated that significant associations exist between social experience scales and mental well-being, as previous studies have established that connecting with others and developing a sense of belonging are key aspects of individuals’ mental well-being (e.g., Bowman, 2010; Kern et al., 2015).

Considering the significance of the academic and social transition process, both in terms of its inherent value and its influence on students’ academic performance and mental well-being, it becomes crucial to possess a reliable tool for assessing these constructs. Hereunder, we critically examine the prevailing trends in conceptualizing and operationalizing students’ transition experiences, shedding light on potential issues and complexities.

## 4. Conceptualizing and operationalizing students’ transition experiences in academic FYHE

This study posits the first-year transition as a ‘period of change’ encompassing the shift from secondary to higher education, that is marked by various challenges experienced by students. Within this context, a process of adaptation can emerge, providing an opportunity for students to navigate and overcome these challenges, ultimately impacting various success outcomes (De Clercq, 2017; Willems, 2022). In transition literature, two “umbrella” constructs are often adopted to describe and examine the nature of first-year students’ academic and social transition experiences, namely academic and social adjustment and integration. These constructs are briefly outlined in the following paragraphs.

### 4.1. First-year students’ adjustment

The most prominent conceptual framework describing students’ *adjustment* during their transition is that of Baker and Siryk (1984, 1986): the student adaptation to college (SAC) model. This framework delineates that the college experience is multifaceted and comprises various “demands” that require a variety of “adjustments” or “coping responses” from the individual (Baker and Siryk, 1986, p. 32). According to the authors, two sets of such demands arise in the academic and social sphere of the HE institution, and the extent to which students effectively adapt to these demands of the new HE environment, is referred to as academic and social adjustment, respectively. The conceptual model continues that both academic and social adjustment each comprise several sub-facets of student experiences, as is detailed in Table 1.

**Table 1:** Sub-facets of academic and social experiences comprehended in conceptualizations of adjustment and integration.

	Academic experiences		Social experiences	
Adjustment	Baker and Siryk (1984, 1986)	Motivation Engagement/effort Effectiveness of studying and academic efforts Satisfaction with academic environment	Baker and Siryk (1984, 1986)	Extent and success of social involvement in general Establishing relationships with other persons on campus Dealing with being away from home and significant persons there (loneliness) Satisfaction with the social aspects of the college environment.
Integration	Review by Hurtado and Carter (1997)	Effort or time spent in activities Students’ perceptions, reported behaviors, and participation in specific activities Students’ satisfaction with aspects of the academic environment Objective performance Combination of the above	Brooman and Darwent (2014)	Belonging to the university community Relationships with old friends Making new friends at university Relationships with family Relationships with staff Establishing relationships through clubs, societies and student union Use of internet in relationships with others Use of mobile phone in relationships with others
	Schaeper (2020)	Self-perceptions of meeting the standards of the HE institution Identification with the normative structure of the academic system Quality of the student-faculty interaction Identification of the individual with the major and enjoyment of studying	Braxton et al. (2000)	Peer-group relationships: (1) Interpersonal relationship with students yielded positive intellectual growth, (2) Having developed close relationships with peers, (3) Support from peers. Out-of-class interactions with faculty: (1) Satisfied with opportunity to meet and interact with faculty, (2) Having developed close relationships with faculty, (3) Non-classroom interactions with faculty had a positive influence on intellectual growth, personal growth, and career goals and aspirations.
	Veldman et al. (2019)	Knowing where to find and how to access university and academic support services Knowing how to prepare for classes and exams Understanding and making use of the university’s academic infrastructure	Torenbeek et al. (2010)	Contact with teachers Contact with fellow students
	Ishitani (2016)	Quantity of student-faculty interaction	Severiens and Wolff (2008)	Interactions among peers (Formal and Informal)

Baker and Siryk’s model is operationalized in a self-report measure: the Student Adaptation to College Questionnaire (SACQ; Baker and Siryk, 1984). In this instrument, academic and social adjustment are measured using several statements (items), each referring to the abovementioned sub-facets of the first-year experience. Respondents are then asked to assess how well they are dealing with that aspect of adjustment.

Today, the SACQ still is the most commonly used instrument to map out first-year student’s academic and social adjustment (e.g., van Rooij et al., 2018b; Watson and Lenz, 2020), and the instrument has proven to be valuable in predicting first-year students’ academic achievement (e.g., review study by Credé and Niehorster, 2012). Nonetheless, questions have been raised about the conceptual foundation of the instrument, as the authors have only published limited information hereupon (see Taylor and Pastor, 2007; Watson and Lenz, 2020). We corroborate this idea that Baker and Siryk (1984, 1986) do not treat in great detail the rationale and theories on which they based their conceptual framework. It is thus unclear where the sub-facets of academic and social adjustment (see Table 1) were derived from, and to what extent they (still) provide a comprehensive representation of the first-year experience nowadays. Taylor and Pastor (2007), who critiqued the SACQ in their work, recommend that future research should first “*pursue further theoretical development of the [adjustment] construct*,” and second, “*either revisit the structure of this instrument or create a new instrument to measure adjustment to college*” (p. 1016).

### 4.2. First-year students’ integration

Research on first-year students’ academic and social *integration* was first outlined in Tinto’s Student Attrition framework (1975). The original works of Tinto (1975, 1993), however, have been critiqued of not having clearly defined “integration” (e.g., Hurtado and Carter, 1997; Braxton, 2000), which has led to various interpretations of the construct, and consequently, a great variety of operational definitions (Hurtado and Carter, 1997). It is clear that, up till today, scholars do not agree on which sub-facets should be incorporated in measures of academic and social integration (Davidson and Wilson, 2013; Lee et al., 2018). To illustrate this latter point, we have summarized in Table 1 the various sub-facets that are considered in different studies, when conceptualizing and operationalizing academic and social integration.

### 4.3. Adjustment and integration: toward conceptual clarity

Due to the vague theoretical grounding of the adjustment construct, and the multitude of interpretations of the integration construct, the overlap of both constructs is ambiguous. Both constructs have been described to be strongly related and are often used interchangeably (e.g., van Rooij et al., 2018a; Larose et al., 2019; Fematt et al., 2021). A few years ago, Tinto defined integration as students’ “*sense of belonging*” during their transition to HE, explaining it is a “*state of being*” based on the students’ perceptions of compatibility with their HE environment (Wolf-Wendel et al., 2009). Building further on this exposition, a sense of belonging, in transition literature, is defined as the psychological sense that one is a valued member of the HE institution community (Hausmann et al., 2007), and has been described to refer to students’ feelings of fitting in, acceptance, and support from the campus community (Strayhorn, 2012). This perspective on integration is in line with the earlier assertion of Braxton (2000) that “*social and academic integration can be viewed as the psychological consequence of interactions with the institutions’ systems*.” (p. 63).

Following this rationale of Braxton (2000) and Tinto [as cited by Wolf-Wendel et al. (2009)], we define *adjustment* as a process of adaptation of behavior and attitudes that may or may not enable a student to effectively meet the various academic and social demands students encounter in the first semester of FYHE. *Integration*, on the other hand, is delineated as the psychological outcome of the adjustment process at a certain point in time. This state of being is based on students’ perception of experiences within the academic and social sphere, reflecting their perceived fit with the new HE environment, and comprising components such as feeling supported, feeling competent, feeling prepared, or feeling related to the chosen study (Braxton, 2000; Wolf-Wendel et al., 2009).

The above described conceptual differentiation between adjustment and integration proved to be valuable for our previous qualitative work (Willems et al., 2021a,b), as it provided two complementary theoretical lenses on the first-year academic and social experience. This difference, however, is less meaningful when operationalizing both constructs in a Likert-type self-report questionnaire that maps out how students are dealing with their social and academic transition at a certain point in the first semester. Indeed, mapping out a student’s level (perceived quality) of academic and social *adjustment* (i.e., the active adaptation of behavior and attitudes as demanded by HE) at a certain point in FYHE, will in effect result in the measurement of this student’s academic and social *integration* (i.e., the psychological state of being that results from an individual’s perception of fit). Let us illustrate this with an example: one facet to which many first-year students will have to actively adjust is the increasing quantity of academic work in HE (Willems et al., 2021a). Measuring where students stand in the process of adapting to a large amount of work, by means of a self-report questionnaire, entails using items that *de facto* elicit students level of perceived competence that they can handle this facet at the time of data collection, such as: “*The amount of work required for the university college is quite manageable*.” In sum, a questionnaire will inevitably elicit the individual’s *perceived* level of adjustment, and thus – according to our theoretical framework – its perceived fit with the new HE environment or integration.

## 5. The present study

Although the above described theoretical difference between adjustment and integration provides some general conceptual clarity, it remains unclear which facets should be considered when examining these multi-faceted concepts. Firstly, the SAC model was developed based on a literature study of student adaptation to college (Baker and Siryk, 1984). It is, however, unclear how the framework offers a comprehensive picture of the first-year experience, as the authors do not detail where the discerned facets of adjustment stem from nor on what basis they were created. Secondly, in the last decades, scholars have given various interpretations to integration, focusing on different aspects of the first-year experience when conceptualizing the construct (see Table 1 for illustrations). This brings to the fore the question which experiences are *most* important according to students themselves in the process of adapting to FYHE, and therefore essential when conceptualizing and operationalizing adjustment and integration.

Moreover, to the best of our knowledge, conceptualizations and quantitative measures of adjustment and integration, up until now, were solely developed in academic HE contexts, leaving the first-year student perspective of professional HE students rather underexplored. Nevertheless, it is clear that this latter, important group of students in professional HE contexts, are confronted with learning environments that are dissimilar to those in academic HE contexts, with regard to its aims, expectations and curricular organization, and it is widely endorsed that the development of theories of the first-year experience should acknowledge the characteristics of such diverse learning environments (e.g., Hearn, 2006; Deil-Amen, 2011).

It is in this context that we developed a new questionnaire, which is based on extensive qualitative empirical research, and which offers a conceptualization of student adjustment and integration that is applicable to a broad range of first-year students in professional HE contexts. More specifically, it describes themes of *critical* academic and social student experiences associated with the transition process in the first semester of professional FYHE, which – adopting the complementary perspective as outlined above – can be regarded as essential facets of academic and social adjustment and integration (see Willems et al., 2021a,b).

With the aim of validating a new self-report questionnaire that provides a comprehensive picture of students’ perceptions of how they are dealing with their social and academic transition at a certain point in the first semester, we translated the unveiled themes of our conceptual framework into a variety of quantitative scales (see Table 2 for a brief description of these 10 academic and 6 social experience themes), which resulted in the creation of the First-year Integration Test (FIT). Indeed, Likert-type self-report questionnaires that aim to map out the adjustment process at a certain point in time, according to our theoretical framework, will inherently always probe for students’ *integration* (as discussed in 3.3 Adjustment and integration: toward conceptual clarity). Consequently, in this study, we provide an instrument for the measurement of the most important aspects of academic and social integration.

**Table 2:** Scales developed for the FIT, description, and item example.

Experience themes/scales	Description	Item example
Academic		
1. Quantity of work	Reflects the extent to which students adjust to the mounting quantity of work in HE.	I feel that we are given too much work for the university college in too little time.- *Reverse coded*
2. General planning semester	Reflects the extent to which students deal with the general planning of the semester (e.g., modular system and its high pace, class schedules, long days).	I have noticed that I can cope well with the general organization of the semester (e.g., schedule, daily structure, module system).
3. Making sense of expectations	Reflects the extent to which students cope with expectations of teachers and the new system of evaluation.	I have a general idea of the questions that we can expect on the examinations.
4. Problems self-regulation	Reflects the extent to which students have problems with self-regulating their academic work (e.g., making a planning, following the planning, evaluating the learning process, organizing oneself, dealing with subject matter efficiently).	I have noticed that I am having trouble scheduling my work for this program.
5. Committing to study	Reflects the extent to which students make an effort and are motivated, with regard to a variety of required tasks related to the study.	I have kept up well with my lessons for this program.
6. Problems following class	Reflects the extent to which students struggle with following class. For instance, adjusting to the high pace of classes, wherein a lot of information is transferred, or to the large class sizes.	I have trouble keeping up during lectures, because the pace is so fast.
7. Taking notes	Reflects the extent to which students deal with taking notes in class.	During lectures, I write down important information in a structured way.
8. Processing learning content	Reflects the extent to which students adjust to autonomously studying the learning contents in HE.	I am able to process the subject matter well.
9. Feeling competent	Reflects the extent to which students feel confident in their own academic capabilities and performances.	I am doing well in this program.
10. Feeling prepared	Reflects the extent to which students feel they acquired necessary skills and important knowledge in secondary education to function well in HE.	My last year in secondary school was a good preparation for the program that I am taking now.
Social		
11. Feelings at the start-off	Reflects how students felt before and during their social transition into HE; the extent to which they looked forward to the transition.	I looked forward to making the transition to the university college, because I would be able to meet new people here.
12. Social self-belief	Reflects the extent to which students feel confident in their capacity of establishing connections with others.	In general, I do not think that I am as good at establishing contacts with other students as other students are.”- *Reverse coded*
13. Establishing first connection	Reflects the extent to which students (initially) create first, rather superficial bonds with their peers.	I have already established initial contacts with new fellow students from the university college.
14. Establishing deep connection	Reflects the extent to which students establish a deeper connection and friendship with their peers.	I have already made good friends at the university college.
15. Feeling supported by peers	Reflects the extent to which students experience help from fellow students when they come across predicaments with regard to their academic work or personal problems.	I feel supported in my studies by my fellow students at the university college.
16. Feelings of loneliness	Reflects the extent to which students feel lonely when they are not able to establish social connections at the university college.	I feel lonesome at the university college.

The following research question is central in this study: *to what extent is the First-year Integration Test (FIT) a valid and reliable instrument to map out the academic and social integration of students in professional HE contexts?* This research question was further specified in four sub-questions:

(a) To what extent does the FIT have good construct validity?
(b) To what extent do the individual scales of the FIT have good internal consistency?
(c) To what extent does the FIT have good convergent and discriminant validity?
(d) To what extent does the FIT have good criterion-related validity with respect to mental well-being and academic achievement?

## 6. Methods

### 6.1. Design and development of the fit questionnaire

In order to identify the most important student experiences of first-year students in professional HE, in two previous studies, qualitative data were collected from 104 purposively selected FYHE students, enrolled in various study programs of a Flemish university college (see Willems et al., 2021a,b). Drawing on the critical incident technique, participants in these studies engaged in the completion of “reflective logs” at the start of the second semester of FYHE. The purpose of these logs was to encourage participants to reflect upon three critical academic experiences and three critical social experiences they encountered during their first semester in HE. Content analyses of the collected narratives unveiled nine main themes of academic integration, and five main themes of social integration (each containing several sub-themes) that university college students perceived to be critical, and which can be considered central constructs that are at play in the multifaceted academic and social transition process in professional HE contexts. Based on the perceived importance of the reported (sub)themes of experiences, we selected 16 (sub)themes (10 academic and 6 social experience themes),^1^ for which we developed items in order to include them in our questionnaire. Table 2 provides a brief description of these 10 academic and 6 social experience themes, as well as an item example that we created to measure each of the themes.

**Figure 1:**
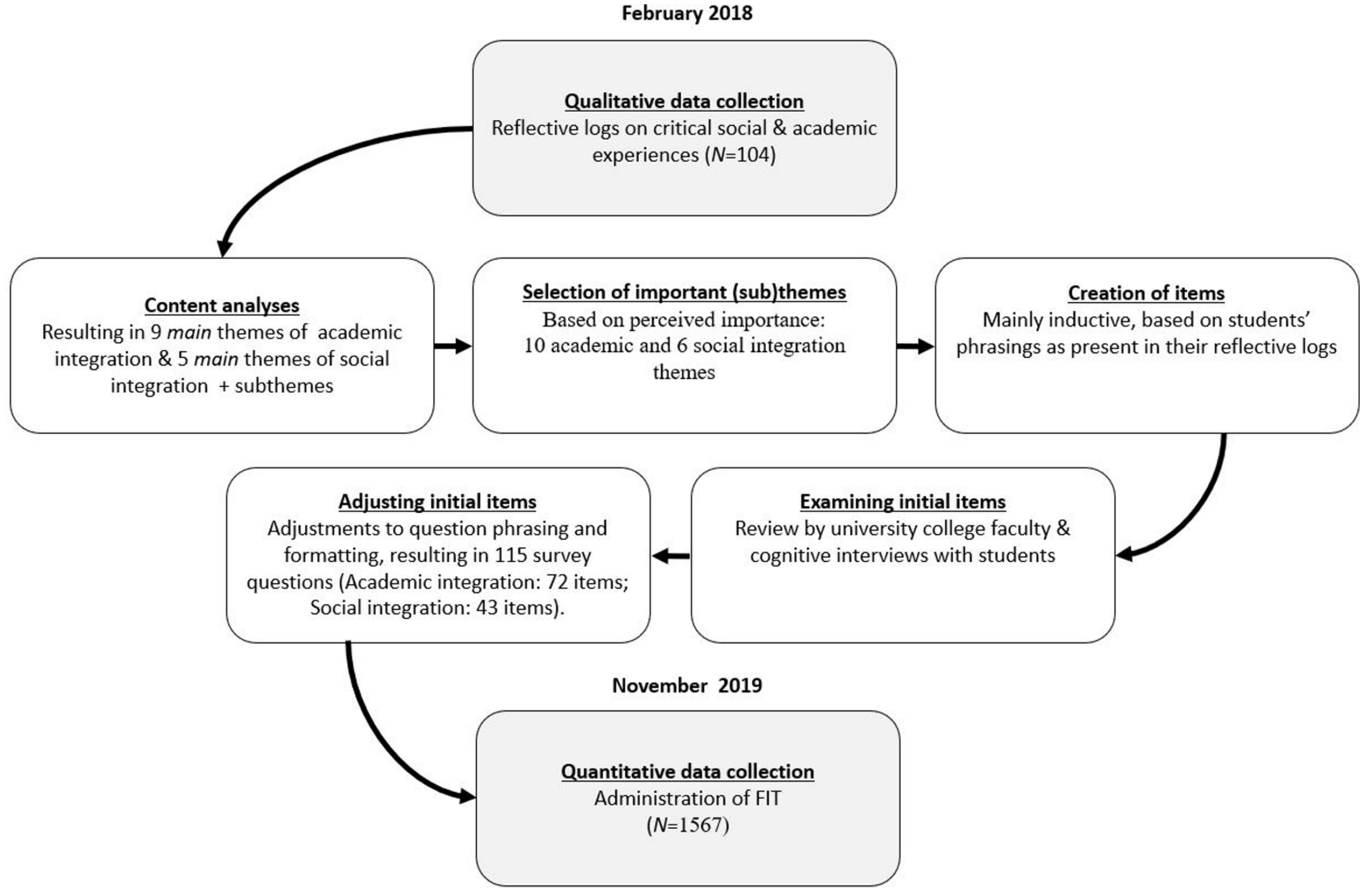
Development of the FIT questionnaire.

For the construction of the items, we mainly used students’ phrasings as present in their reflective logs. In addition, for the formulation of the items of two of these scales, we also drew inspiration from existing items; four of the items of the ‘*Feeling prepared*’ scale were based on the work of Torenbeek et al. (2010), and five of the ‘*Committing to the study*’ scale, were based on items from the SACQ (Baker and Siryk, 1984, 1986). In order to further enhance face and construct validity (Cohen et al., 2011), this list of preliminary items was reviewed by members of the university college faculty and by four second-year university college students. After making several adjustments to both question phrasing and formatting, this resulted in the final questionnaire, counting 115 survey questions in total (Academic experience: 72 items; Social experience: 43 items; see Appendices A and B). All item are scored on a five-point Likert scale ranging from ‘Completely disagree’ to ‘Completely agree’. An overview of the steps in the development process of the FIT questionnaire is provided in Figure 1.

### 6.2. Participants, procedure, and additional measures

This first version of the FIT was administered in November (approximately two months after the start of the first semester) of academic year 2019–2020 in a Flemish university college. In total, 2,583 students completed the questionnaire. From this initial dataset, we omitted students who had indicated that they had already studied at a university or university college (*N* = 840), who did not gave their informed consent (*N* = 113), and students who did not answer correctly on any of the four control items as indicator of careless responding (*N* = 63), which brought about our final dataset of 1,567 respondents. These students were enrolled in 17 different professional bachelor programs (see Appendix A). To examine the validity of the questionnaire, we randomly split the sample in a calibration (*N* = 783) and a validation sample (*N* = 784).

In order to examine the criterion-related validity of the FIT scales (RQ1d), we also collected data regarding the respondents’ mental well-being and academic achievement. Firstly, two weeks after administering the initial version of the FIT, we administered the validated Warwick-Edinburgh Mental Well-being Scale (WEMWBS; Tennant et al., 2007). This 14 items scale is scored on a 5-point Likert scale ranging from ‘none of the time’ to ‘all of the time’ (e.g., “I’ve been feeling optimistic about the future”; *α* = 0.89). The development of the WEMWBS scale was driven by the objective of capturing a comprehensive understanding of well-being, incorporating affective-emotional aspects, cognitive-evaluative dimensions, and psychological functioning, which corroborates with our definition of mental well-being. Drawing upon previous scales, the WEMWBS was designed in a concise format to facilitate its effective utilization in population-level surveys (Tennant et al., 2007). It should be noted that only 134 respondents completed the well-being scale, 52 of which could be linked to the 1,567 students that were included in the final FIT dataset.^2^

Secondly, students’ academic achievement data were collected through the administration database of the university college involved. Academic achievement was conceptualized as early Grade Point Average (GPA): the weighted average score across all examinations after the first semester.

### 6.3. Analysis

With the intention of reducing the complexity of the analyses, we examined the two sets of items pertaining to academic and social experiences separately. To address our first research question (RQ1a) regarding the factor structure of the survey, we initially examined each individual scale. Starting with the calibration sample, we conducted an exploratory factor analysis (EFA) with oblique rotation for each scale to explore their dimensionalities. This allowed us to determine whether each initially developed scale indeed measured a single component. To validate our findings, we performed confirmatory factor analysis (CFA) on each scale using the calibration sample. Subsequently, we conducted exploratory and confirmatory factor analyses on the calibration sample again, but this time we examined the two comprehensive models: (1) the set of 10 academic experience scales and (2) the set of 6 social experience scales (see Table 2).

In this phase of the analyses, modifications were made to the structure of the factor models and malfunctioning or inferior items were deleted. For the deletion of items, we simultaneously used several criteria: (1) in the factor analysis the item loads to another factor than was theoretically assumed, (2) the item has a relatively low factor loading; (3) the removal of an item enhances the Cronbach’s alpha index with minimum 0.01, (4) the item has a corrected item-total correlation of under 0.30 (Nunnally and Bernstein, 1994). For every decision to remove an item, theoretical arguments and goodness-of fit indices of the CFA’s were considered as well.

To compare the fit of different CFA models, we utilized the Akaike Information Criterion (AIC). A lower AIC value indicates a better fit compared to a model with a higher AIC value (Kline, 2016). Additional fit indices used to assess model fit included the comparative fit index (CFI), root mean square error of approximation (RMSEA), and standard root mean square residual (SRMR) (MacCallum et al., 1996; Hu and Bentler, 1999).

In the next step, we estimated the resulting model on the validation sample to ensure that the modifications were not based on chance, as they were made using a specific calibration sample (MacCallum et al., 1992). We first examined each scale separately and then estimated the full CFA models with all scales included.

Following the previous analyses, we proceeded to calculate Cronbach’s alphas for the resulting scales in both the calibration and validation samples. This step aimed to assess the internal consistency of the individual scales within the FIT, addressing our second research question (RQ1b). To determine the convergent and discriminant validity of the scales (RQ1c), we examined the correlations between the scales under investigation. These correlation analyses were conducted using the entire sample consisting of 1,567 participants. Finally, in order to examine the criterion-related validity of the different first-year integration scales (RQ1d), we calculated Pearson’s moment correlations between every academic and social integration variable on the one hand, and mental well-being and academic achievement on the other hand. Once again, this analysis was performed using the entire sample.

## 7. Results

### 7.1. Construct validity

**Figure 2:**
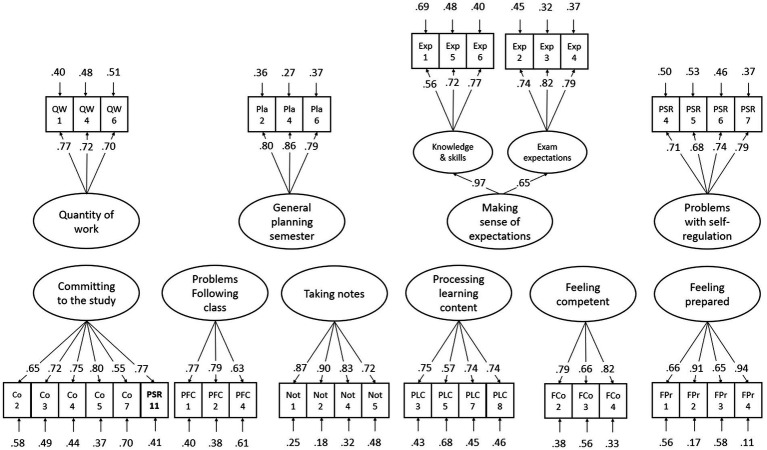
Factor structure and standardized parameter estimates of the final academic CFA-model, fitted on validation sample.

Several alterations were carried out at the scale level to obtain parsimonious models with good model fit, of which the different scales have good internal consistencies. Firstly, in the *academic* integration scales, a total of 32 items were deleted following the criteria and procedure as outlined in the analysis section above. Furthermore, EFA analyses made clear that item PSR11 (“*I have not been very efficient in using my study time recently*”), which was initially included under the “*Problems with self-regulation*” scale, is actually perceived by students to belong to “*Committing to the study*” When subjected to factor analysis in the calibration sample, this item exhibited a *substantial* factor loading of 0.77 on the “Committing to the study” factor. In contrast, its factor loading on the “Problems with self-regulation” factor was notably low, measuring only −0.02. This reclassification was also theoretically justifiable. Next, EFA’s on both the calibration and validation sample showed that the scale “*Making sense of expectations*” comprised two sub-scales: one sub-scale (“*Knowledge & skills*” in Figure 2) refers to the extent to which students have an idea of the knowledge and skills that is expected of them, the other (“*Exam expectations*” in Figure 2) refers to the extent to which students know what questions they can expect on examinations. Figure 2 shows the resulting CFA model for the academic section of the FIT (Calibration sample: CFI = 0.946, RMSEA = 0.041, SRMR = 0.046; Validation sample: CFI = 0.944, RMSEA = 0.041, SRMR = 0.046). All latent factors in the model were allowed to correlate, but for reasons of clarity, these intercorrelations were omitted from the figure. Manifest correlations between all academic constructs, however, can be found in Table 3. The final set of academic FIT items are presented in Appendix B.

**Table 3:** Intercorrelations between academic and social integration variables.

	1.	2.	3.	4.	5.	6.	7.	8.	9.	10.	11.	12.	13.	14.	15.	16.
Academic																
1. Quantity of work	1															
2. General planning semester	0.32^**^	1														
3. Making sense of expectations	0.33^**^	0.26^**^	1													
4. Problems self-regulation	−0.36^**^	−0.38^**^	−0.34^**^	1												
5. Committing to study	0.10^**^	0.14^**^	0.11^**^	−0.60^**^	1											
6. Problems following class	−0.43^**^	−0.37^**^	−0.31^**^	0.42^**^	−0.16^**^	1										
7. Taking notes	0.10^**^	0.16^**^	0.12^**^	−0.36^**^	0.39^**^	−0.23^**^	1									
8. Processing learning content	0.36^**^	0.37^**^	0.39^**^	−0.58^**^	0.37^**^	−0.52^**^	0.35^**^	1								
9. Feeling competent	0.24^**^	0.34^**^	0.25^**^	−0.51^**^	0.42^**^	−0.33^**^	0.27^**^	0.59^**^	1							
10. Feeling prepared	0.05^*^	0.06^*^	0.07^*^	−0.11^**^	0.11^**^	−0.11^**^	0.14^**^	0.17^**^	0.20^**^	1						
Social																
11. Feelings at start-off	0.08^**^	0.19^**^	0.08^**^	−0.09^**^	0.12^**^	−0.08^**^	0.07^**^	0.14^**^	0.16^**^	0.09^**^	1					
12. Social self-belief	0.10^**^	0.11^**^	0.08^**^	−0.15^**^	0.13^**^	−0.15^**^	0.08^**^	0.22^**^	0.22^**^	0.08^**^	0.43^**^	1				
13. Establishing first connection	0.06^*^	0.25^**^	0.07^**^	−0.15^**^	0.13^**^	−0.10^**^	0.12^**^	0.16^**^	0.24^**^	0.05	0.28^**^	0.40^**^	1			
14. Establishing deep connection	0.02	0.19^**^	0.07^**^	−0.15^**^	0.13^**^	−0.01	0.10^**^	0.13^**^	0.21^**^	0.08^**^	0.31^**^	0.40^**^	0.61^**^	1		
15. Feeling supported (peers)	0.12^**^	0.21^**^	0.15^**^	−0.19^**^	0.18^**^	−0.11^**^	0.16^**^	0.20^**^	0.23^**^	0.06^*^	0.26^**^	0.29^**^	0.53^**^	0.62^**^	1	
16. Feeling lonely	−0.13^**^	−0.27^**^	−0.11^**^	0.26^**^	−0.17^**^	0.12^**^	−0.12^**^	−0.19^**^	−0.25^**^	−0.07^*^	−0.24^**^	−0.45^**^	−0.56^**^	−0.61^**^	−0.53^**^	1

**p < 0.01; *p < 0.05.

**Figure 3:**
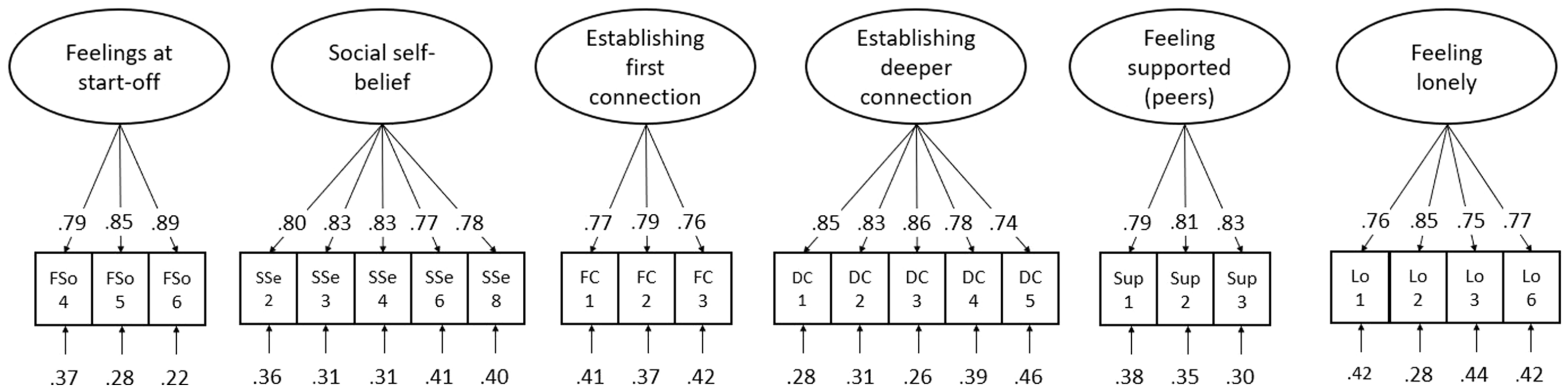
Factor structure and standardized parameter estimates of the final social CFA-model, fitted on validation sample.

Figure 3, then, shows the resulting CFA model for the *social* integration scales of the FIT (Calibration sample: CFI = 0.958, RMSEA = 0.050, SRMR = 0.038; Validation sample: CFI = 0.960, RMSEA = 0.052, SRMR = 0.033). Again, the intercorrelations between factors were omitted from the figure, and manifest correlations between constructs are displayed in Table 3. In total, 20 items were appointed to be inferior, and thus were deleted. The final set of social FIT items are presented in Appendix C.

Finally, we also fitted all resulting social (6) and academic (10) scales together in one CFA model. This full model had good fit in both the calibration (CFI = 0.940; RMSEA = 0.034; SRMR = 0.039) and validation (CFI = 0.945; RMSEA = 0.033; SRMR = 0.039) sample. In summary, subsequent to the removal of inadequate items, the FIT exhibits robust construct validity, thereby confirming its ability to effectively measure the constructs outlined in the original theoretical framework.

### 7.2. Reliability

The alpha coefficients in Table 4 indicate that every scale in both calibration and validation samples is reliable to highly reliable, ranging from *α* = 0.71 to *α* = 0.90 (Cohen et al., 2011).

**Table 4:** Internal consistencies of academic and social integration scales, within calibration and validation sample.

	# items	α_Calibration_	α_Validation_
Academic			
Quantity of work	3	0.78	0.77
General planning semester	3	0.84	0.85
Making sense of expectations	6	0.83	0.81
Knowledge and skills	3	0.76	0.71
Exam expectations	3	0.85	0.83
Problems self-regulation	4	0.83	0.82
Committing to study	6	0.86	0.85
Problems following class	3	0.76	0.76
Taking notes	4	0.89	0.90
Processing learning content	4	0.81	0.78
Feeling competent	3	0.82	0.79
Feeling prepared	4	0.88	0.87
Social			
Feelings at start-off	3	0.86	0.88
Social self-belief	5	0.89	0.90
Establishing first connection	3	0.80	0.82
Establishing deep connection	5	0.88	0.90
Feeling supported (peers)	3	0.83	0.85
Feeling lonely	4	0.81	0.85

### 7.3. Convergent and discriminant validity

The correlations between the variables under study, as displayed in Table 3, all have the anticipated directions, which adds to the convergent and discriminant validity of the scales. For the academic scales, for instance, *Problems with self-regulation* is, as expected, significantly related to all other academic integration scales. In this light, *Feeling prepared* was weakly (Cohen, 1988) correlated with *problems with self-regulation* (*r* = −0.11). Furthermore and as theoretically expected, moderate correlations were found between *Problems with self-regulation* and dealing with a mounting *Quantity of work* (*r* = −0.36), *General planning semester* (*r* = −0.38), *Making sense of expectations* (*r* = −0.34), *Problems following class* (*r* = 0.42), and *Taking notes* (*r* = −0.36). This is also the case for the strong relationships between *Problems with self-regulation* and the remaining academic scales: *Committing to study* (*r =* −0.60), *Processing learning content* (*r* = −0.58), and *Feeling competent* (*r* = −0.51).

With regard to the social integration scales, all scales are significantly related to each other in meaningful ways. For example, *Loneliness* is negatively correlated to all other social integration scales; a weak relationship was found between loneliness and *Feelings at start-off* (*r* = −0.24), while moderate to strong relationships emerged between loneliness and *Social self-beliefs* (*r* = −0.45), *Establishing first connection* (*r* = −0.56), *Establishing deep connection* (*r* = −0.61), and *Feeling supported by peers* (*r* = −0.53).

### 7.4. Criterion-related validity

To assess the criterion-related validity of the academic and social integration scales, we calculated Pearson’s moment correlations between each of these variables and mental well-being and GPA after the first semester of FYHE (Table 5).

**Table 5:** Pearson’s correlations between all scales and mental well-being and early GPA.

	Well-being	Early GPA
	*N* = 50–52	*N* = 1,407–1,470
Academic		
Quantity of work	0.26	0.04
General planning semester	0.48^**^	0.12^**^
Making sense of expectations	0.42^*^	0.06^*^
Knowledge and skills	0.50^**^	0.02
Exam expectations	0.17	0.07^**^
Problems self-regulation	−0.46^**^	−0.20^**^
Committing to study	0.24	0.23^**^
Problems following class	−0.04	−0.14^**^
Taking notes	−0.07	0.09^**^
Processing learning content	0.32^*^	0.24^**^
Feeling competent	0.58^**^	0.33^**^
Feeling prepared	0.28^*^	0.13^**^
Social		
Feelings at start-off	0.39^**^	0.04
Social self-belief	0.60^**^	−0.04
Establishing first connection	0.44^**^	0.13^**^
Establishing deep connection	0.36^**^	0.01
Feeling supported (Peers)	0.42^**^	0.03
Feeling lonely	−0.51^**^	0.00

**p < 0.01, *p < 0.05.

Even though a small sample of students completed the mental well-being scale (WEBWBS), several significant relationships with the FIT scales were found. Firstly, all social integration scales have moderate to strong (Cohen, 1988) relationships with mental well-being: dealing with unknown (*r* = 0.39), social self-beliefs (*r* = 0.60), establishing first connection (*r* = 0.44), establishing deep connection (*r* = 0.36), feeling supported (*r* = 0.42), loneliness (*r* = −0.51). With regard to the academic integration scales, then, mental well-being was moderately to strongly correlated to general planning semester (*r* = 0.48), making sense of expectations – knowledge & skills (*r* = 0.50), problems with self-regulation (*r* = −0.46), processing learning content (*r* = 0.32), and feeling competent (0.58). A weak significant correlation was found between mental well-being and feeling prepared (*r* = 0.28).

Further, most academic integration scales were significantly related to students’ early academic achievement. Weak correlations were found between early GPA and general planning semester (*r* = 0.12), making sense of expectations – Exam expectations (*r* = 0.07), problems with self-regulation (*r* = −0.20), committing to study (*r* = 0.23), problems following class (*r* = −0.14), taking notes (*r* = 0.09), processing learning content (*r* = 0.24), feeling prepared (*r* = 0.13). Feeling competent was moderately correlated with early GPA (*r* = 0.33). The only significant association between early GPA and the social integration scales was the weak correlation with establishing first connection (*r* = 0.13).

## 8. Discussion and conclusion

This study commenced with a thorough literature review, revealing that scholars generally do not agree on which important facets should be incorporated in quantitative measures of the academic and social first-year experience (e.g., Davidson and Wilson, 2013; Lee et al., 2018). Furthermore, such scales are typically developed in academic HE contexts, and are rather disconnected from the experiences of students in more professional HE programs. In this light, the First-year integration test (FIT) is an instrument that aims to map out the most important academic and social experiences of first-year students in professional HE during their transition, and which design was based on thorough qualitative analyses of the reported ‘critical incidents’ of 104 purposively selected FYHE students in professional HE programs (Willems et al., 2021a,b). In the present study, the psychometric value of the FIT was investigated.

Exploratory and confirmatory factor analyses on both a calibration and a validation sample show that, after the deletion of several inferior items, the FIT has good construct validity, indicating that the resulting instrument conforms to the theoretical framework that was initially proposed (Cohen et al., 2011). Furthermore, the high Cronbach’s alpha values of the resulting scales of the FIT demonstrate that they are all internally consistent.

Next, the present study has provided evidence for the convergent and discriminant validity of the instrument, as the academic as well as the social integration scales are all related to each other in meaningful ways. Indeed, as the constructs measured by the present study relate to widely-used concepts in transition literature that were developed in academic HE contexts, it is possible to compare these correlations to the findings of previous research. With regard to the academic experience variables, for instance, it was to be expected that ‘problems with self-regulation’ would negatively relate to ‘processing the learning content’ and ‘feeling competent’ (e.g., Schunk and Zimmerman, 2012; Willems et al., 2019). Another example here, is the expected positive relation between ‘feeling prepared’ and ‘feeling competent’ (Noyens et al., 2020). With reference to the social experience variables, we hypothesized, for instance, that loneliness would be negatively related to all other social variables (e.g., Lee and Goldstein, 2016).

Finally, our results also show that the academic and social integration scales – with the exception of “*Dealing with the quantity of work*” – all have criterion related validity with regard to mental well-being and/or academic achievement. In this light and in line with previous research, we found that, most of the academic integration scales are significantly and meaningfully related to academic achievement (e.g., Robbins et al., 2004; Richardson et al., 2012). Moreover, many of the academic integration scales are also meaningfully related to students’ mental well-being.

Our results further show that the social experience variables are primarily related to students’ mental well-being. This observation is not unexpected, as prior research has established the fundamental role of interpersonal connections and a sense of belonging in fostering individuals’ mental well-being (e.g., Bowman, 2010; Kern et al., 2015). Next, we also want to corroborate the call for more (qualitative) research into the relationship between the first-year social experience and academic achievement (e.g., De Clercq et al., 2018). Indeed, the findings of previous studies on the relationship between achievement and social integration are inconclusive (e.g., Robbins et al., 2004; Richardson et al., 2012; Davidson and Wilson, 2013). In light of our study, we believe it to be fruitful to further examine why our results showed a positive relationship between *Establishing first connection* and academic achievement, but no such relationship was found between *Feeling supported by peers* and academic achievement.

### 8.1. Limitations and future work

A first important limitation is that, although multiple study programs were involved and a substantial sample of students participated, this validation study was carried out in only one university college. More work, that deploys the FIT in other HE contexts, will need to be done to determine whether the instrument is also transferable to other professional HE education settings. Additionally, it would also be valuable to explore the potential applicability of the FIT scales across academic HE contexts. The aim of this study was to develop a widely applicable questionnaire, capable of *comprehensively* capturing the most important aspects of first-year students’ academic and social integration in professional HE contexts. To achieve this, our prior qualitative research took a broader, more remote perspective, leading to the identification and subsequent operationalization of rather *abstract* and *general* themes within the FIT. Thus, the FIT scales do not specifically focus only on the professional aspects of HE programs, which might suggest that the instrument’s psychometric value may extend to university contexts as well.

Further, it is also important to bear in mind that we were only able to examine the relationship between academic and social experience variables and fist-year students’ mental well-being (measured two weeks after FIT scales), using data of a very small group (*N* = 52) of respondents. This low sample size notwithstanding, we did find several significant relationships, giving us first indications that the variables under study indeed have considerable predictive value with regard to students’ mental well-being in the first semester of FYHE. Future research, however, should further examine these relationships, using a more substantive data set.

Thirdly, it should be mentioned that the validation study was carried out cross-sectionally, at only one point in the first semester (in November, approximately two months after the start of the first semester). Taking into account the tumultuous nature of the first semester (e.g., Coertjens et al., 2017), the question could be raised whether the factor structure and relationships found in this study, would remain stable throughout the complete FYHE (and beyond). To enhance the instrument's robustness and deepen our understanding of its impact, future research designs should incorporate multiple measurement moments throughout the FYHE experience.

### 8.2. Practical implications

Despite the limitations mentioned above, we believe that the FIT in its current form, counting a feasible set of 63 items (Academic: 40 items, social: 23 items), is valuable not only for research but also for practice. To begin with, its design based on actual first-year students’ voices, the FIT provides scholars with an empirically-based and comprehensive research instrument to assess the most important integration experiences that are central to professional FYHE students’ academic and social transition process, in a valid and reliable fashion. This, for instance, allows for large-scale measurement of the concepts that were previously unveiled in our qualitative work.

The FIT might also be a valuable tool in the quality assurance system of (professional) HE institutions, when used to monitor critical aspects of the first-year students’ academic and social experience (Parpala and Lindblom-Ylänne, 2012). Indeed, certain aspects of integration could be *more* challenging for students in specific study programs. Examining these kind of data, thus, can provide important evidence for assessing the quality of the first-year student experience in the HE institution. In light of this, it is important to note that the FIT should not be employed as a definitive measure to assess the overall quality of education in an institution, since it is based solely on students’ perceptions of integration. Rather, the FIT serves as a valuable tool for monitoring the first-year student population and identifying potential problematic situations. Finally, the development of the FIT might answer to the call of developing instruments for student *feedback generation* that aim to increase first-year students’ self-knowledge with regard to essential facets of their transition to HE (Kyndt et al., 2017). Questionnaire-based feedback, when implemented in an institutional student guidance system that offers systematic opportunities for student counseling, can be an important vehicle for student reflection, and might ultimately facilitate students’ transition to HE (Ruohoniemi et al., 2017).

As a final remark, scholars and practitioners should be aware of a potential drawback, induced by the FIT’s current item count, namely the possibility of inducing respondent fatigue. With 63 items to complete, students may experience fatigue and a decrease in their engagement and attention when providing responses. This could impact the accuracy and quality of the data collected (Cohen et al., 2011). On the upside, however, the inclusion of a larger number of items allowed for a more comprehensive and qualitative measurement of the complex concept of integration, that encompasses various sub-concepts. The thoroughness and depth of the instrument in its current form might offer valuable insights that can inform both research and practical interventions aimed at supporting students’ successful integration into higher education.

## Data availability statement

The raw data supporting the conclusions of this article will be made available by the authors, without undue reservation.

## Ethics statement

The studies involving human participants were reviewed and approved by Ethics Committee for the Social Sciences and Humanities (EASHW) – University of Antwerp. The patients/participants provided their written informed consent to participate in this study.

## Author contributions

JW provided initial conception, organization, main writing of the text, analyzed the data, prepared all figures, and tables. VV was involved in data collection and acted as consultant for the construction of the items of the First-year Integration Test. LC and VD contributed to the construction of the FIT questionnaire, to the conception of the research design, and to data analysis and text writing. All the authors contributed to the article and approved the submitted version.

## Funding

This work was supported by a grant from the Internal Research fund of the University of Antwerp (BOF-DOCPRO4).

## Conflict of interest

The authors declare that the research was conducted in the absence of any commercial or financial relationships that could be construed as a potential conflict of interest.

## Publisher’s note

All claims expressed in this article are solely those of the authors and do not necessarily represent those of their affiliated organizations, or those of the publisher, the editors and the reviewers. Any product that may be evaluated in this article, or claim that may be made by its manufacturer, is not guaranteed or endorsed by the publisher.

## Footnotes

^1^The disbalance in academic and social themes arises from the qualitative analysis of the collected narratives, wherein we uncovered a greater number of academic (sub)themes compared to social (sub)themes.

^2^Firstly, the coupling of the two datasets relied on self-reported student numbers. Student numbers of 62 of the 134 respondents who completed the well-being questionnaire could not be matched to student numbers reported in the FIT dataset (either respondents did not participate in this latter data collection, or they reported incorrect student numbers). Secondly, 20 of the 72 remaining respondents that could be matched to the FIT- questionnaire, did not give their informed consent.
