# Supplementary material for: The First-year Integration Test: a validation study
Source: Front Psychol. 2023 Aug 10;14:1101234. doi: 10.3389/fpsyg.2023.1101234 (PMC10448899; doi:10.3389/fpsyg.2023.1101234)
Supplement: Supplementary file 1 [file Data_Sheet_1.docx]

Appendices

*Appendix A*

Table A1. Student numbers per study programme

| **Study programme** | ***N*** |
| --- | --- |
| Business management | 281 |
| Secondary education | 167 |
| Nursing | 163 |
| Graphic and digital media | 154 |
| Social work | 114 |
| Office management | 97 |
| Speech language therapy | 90 |
| Occupational therapy | 90 |
| Preschool education | 78 |
| Midwifery | 69 |
| Early childhood education | 59 |
| Communication management | 54 |
| Journalism | 50 |
| Dental hygiene | 48 |
| Podiatry | 41 |
| Audiology | 9 |
| Primary education | 3 |

Appendix B

**Table B1**. Items first-year academic experience

| **Quantity of work** | |
| --- | --- |
| QW1 | I feel that we are given too much work for the university college in too little time. (-) |
| QW2 | *I am coping well with the work pressure at the university college.*^1^ |
| QW3 | *The work for the university college is piling up too fast for me. (-)* |
| QW4 | The amount of work required for the university college is quite manageable. |
| QW5 | *I do not think that I have too much work for the university college.* |
| QW6 | I feel that the lecturers are giving us too many assignments and subject matter to process. (-) |
| **General planning of the semester** | |
| Pla1 | *I have quickly adjusted to the general organisation of the semester (e.g., schedule, daily structure, module system).* |
| Pla2 | It is still difficult for me to get used to the general organisation of the semester (e.g., schedule, daily structure, module system). (-) |
| Pla3 | *The general organisation of the first semester (e.g., schedule, daily structure, module system) here at the university college fits me well.* |
| Pla4 | I am having trouble with the general organisation of this semester (e.g., schedule, daily structure, module system). (-) |
| Pla5 | *The general organisation of the first semester (e.g., schedule, daily structure, module system) suits me.* |
| Pla6 | I have noticed that I can cope well with the general organisation of the semester (e.g., schedule, daily structure, module system). |
| **Making sense of expectations in the new learning environment** | |
| Exp1 | It is not clear what the lecturers at the university college expect me to know and be able to do. (-) |
| Exp2 | It is difficult for me to anticipate what the questions on the examinations will look like. (-) |
| Exp3 | I have a general idea of the questions that we can expect on the examinations. |
| Exp4 | I know what I should expect from the examinations. |
| Exp5 | I already have an idea of what the lecturers expect of me with regard to what I should know and be able to do. |
| Exp6 | It is clear to me what I should know and be able to do. |
| **Problems with self-regulation** | |
| PSR1 | *When I have set a schedule, I have trouble sticking to it.* |
| PSR2 | *I have trouble meeting deadlines in this programme.* |
| PSR3 | *It is difficult for me to estimate how much time I should spend on my work for this programme.* |
| PSR4 | I have noticed that I am having trouble scheduling my work for this programme. |
| PSR5 | I have absolutely no idea of how I can keep up with my work for this programme. |
| PSR6 | I am having trouble organising my work for this programme well. |
| PSR7 | The organisation of my work for the programme is not going smoothly at all. |
| PSR8 | *I do not have a good idea of how I should set up a study schedule.* |
| PSR9 | *To be honest, I have no idea of what would be a good way to approach my studies.* |
| PSR10 | *I have trouble making adjustments when I notice that my schedule for this programme is not in order.* |
| PSR11 | ~~I have not been very efficient in using my study time recently. (-)~~ ^2^ |
| **Committing to the study** | |
| Co1 | *I prepare well for lectures when I have to.* |
| Co2 | I have kept up well with my lessons for this programme. |
| Co3 | I have not been very motivated to study recently. (-) |
| Co4 | I have a lot of trouble getting started with studying. (-) |
| Co5 | I am not working as hard for my studies as I should. (-) |
| Co6 | *I am trying very hard to miss as few lectures as possible.* |
| Co7 | I am working hard enough on the assignments for this programme. |
| **Problems with Following class** | |
| PFC1 | I have trouble keeping up during lectures, because the pace is so fast. |
| PFC2 | A very large volume of subject matter is covered during lectures, and this sometimes makes it difficult for me to keep up. |
| PFC3 | *I am having trouble adjusting to the style of teaching in my current programme.* |
| PFC4 | I am currently having more trouble keeping up with the lectures than I previously did in secondary school. |
| PFC5 | *It is difficult for me to concentrate for such a long time during lectures.* |
| **Taking notes** | |
| Not1 | During lectures, I write down important information in a structured way. |
| Not2 | I take clear notes during lectures. |
| Not3 | *I clearly note important aspects during lectures.* |
| Not4 | At the end of the lecture, the important aspects are clearly stated in my notes. |
| Not5 | I am not good at taking notes during lectures. (-) |
| Not6 | *I am having trouble taking clear notes during lectures. (-)* |
| **Processing learning content** | |
| PLC1 | *It is easy for me to process the subject matter.* |
| PLC2 | *I have noticed that my manner of studying is not working well. (-)* |
| PLC3 | I am able to process the subject matter well. |
| PLC4 | *I am having a lot of trouble processing the subject matter. (-)* |
| PLC5 | I have no trouble summarising the subject matter on my own. |
| PLC6 | *Processing the subject matter is difficult. (-)* |
| PLC7 | Rehearsing the subject matter is going smoothly. |
| PLC8 | I have trouble processing the subject matter on my own. (-) |
| PLC9 | *I am quite successful in separating the important aspects of the subject matter from the less important aspects.* |
| **Feeling competent** | |
| FCo1 | *I would like to do better in this programme. (-)* |
| FCo2 | I am doing well in this programme. |
| FCo3 | My performance in this programme is often poor. (-) |
| FCo4 | I am satisfied with my performance in this programme. |
| FCo5 | *I am afraid that this programme will be too difficult for me. (-)* |
| FCo6 | *I think that I will be able to manage this programme well.* |
| FCo7 | *I am very satisfied with my studying situation in this programme.* |
| FCo8 | *I feel that I am under a lot of pressure to do well in this programme. (-)* |
| FCo9 | *I am constantly feeling that I am lagging behind in comparison to my fellow students. (-)* |
| FCo10 | *I am on the right track in this programme.* |
| **Feeling prepared** | |
| FPr1 | My last year in secondary school was a good preparation for the programme that I am taking now. |
| FPr2 | The courses in my current programme clearly build on the programme that I took in secondary school. |
| FPr3 | There is a lot of overlap between secondary school and my programme in higher education. |
| FPr4 | I feel that the teaching content in my current programme corresponds very closely to the teaching content from secondary school. |
| FPr5 | *I learned many of the skills that I need in order to do well in my current programme when I was in secondary school.* |
| FPr6 | *The manner in which I should work in my current programme is the same as it was in secondary school.* |

*Notes: (1) Items printed in italics were later, based on reliability and factor analysis, removed.; (2) Analyses showed that PSR11 is an indicator of Committing to the study rather than of Problems with self-regulation*

Appendix C

**Table C1**. Items first-year social experience

| **Feelings at the start-off** | |
| --- | --- |
| FSo1 | *The prospect of having to get to know new people at the university college was a source of considerable stress for me. (-)* |
| FSo2 | *It was difficult for me to leave the familiar social environment of my secondary school behind me. (-)* |
| FSo3 | *When I came to the university college, I was afraid that I would not make any new friends. (-)* |
| FSo4 | I looked forward to making the transition to the university college, because I would be able to meet new people here. |
| FSo5 | When I came to the university college, I looked forward to becoming immersed in a new group of people. |
| FSo6 | The prospect that I would get to know new people at the university college made me happy. |
| **Social self-belief** | |
| SSe1 | *In general, I think that I can make friends quickly.* |
| SSe2 | In general, I do not think that I am as good at establishing contacts with other students as other students are. (-) |
| SSe3 | In general, I am satisfied with my social skills. |
| SSe4 | I am confident that I can make friends quickly in most situations. |
| SSe5 | *When I see someone whom I would like to meet, I usually have the confidence to approach that person.* |
| SSe6 | In general, I think that I can get along fine in social gatherings. |
| SSe7 | *When I meet someone interesting but have trouble making friends, I am usually quick to stop trying to make further contact. (-)* |
| SSe8 | In general, I do not think that my social skills are good enough. (-) |
| **Establishing a first connection** | |
| FC1 | I have already established initial contacts with new fellow students from the university college. |
| FC2 | I have already gotten to know a number of new fellow students here at the university college. |
| FC3 | I have already made some acquaintance with a number of new fellow students at the university college. |
| FC4 | *It is not easy to get to know my new fellow students at the university college. (-)* |
| FC5 | *It is difficult for me to get to know new fellow students at the university college. (-)* |
| FC6 | *I have not been very successful in establishing contact with new fellow students at the university college. (-)* |
| FC7 | *I feel that I do not belong anywhere at the university college. (-)* |
| **Establishing a deeper connection** | |
| DC1 | I have already made good friends at the university college. |
| DC2 | I am part of a close-knit group of fellow students. |
| DC3 | Strong friendships have formed with my fellow students. |
| DC4 | I feel a strong connection to my fellow students. |
| DC5 | The ties to my fellow students have become so close that we often do fun things together outside of school hours. |
| DC6 | *The ties that I have developed with fellow students are much less shallow than they were at the beginning of the semester.* |
| **Feeling supported (peers)** | |
| Sup1 | I feel supported in my studies by my fellow students at the university college. |
| Sup2 | I know that I can count on my fellow students when I am having trouble in my studies. |
| Sup3 | My fellow students and I help each other with our studies whenever we can. |
| Sup4 | *My fellow students are a major source of support in my studies.* |
| Sup5 | *I do not feel that I can turn to my fellow students when I am having trouble with my studies. (-)* |
| Sup6 | *I would like to have more help from my fellow students with regard to my studies. (-)* |
| Sup7 | *Some fellow students at the university college are very helpful when I am having a hard time.* |
| Sup8 | *I would like to feel more supported by some of my fellow students with regard to personal aspects. (-)* |
| Sup9 | *I can turn to several fellow students when I am having personal problems.* |
| Sup10 | *I can rely on my fellow students when I want to discuss personal matters.* |
| **Feeling lonely** | |
| Lo1 | I am often alone at the university college. |
| Lo2 | I feel lonesome at the university college. |
| Lo3 | I do not know very many people at the university college, and this makes me feel that I am alone. |
| Lo4 | *I do not have much contact with fellow students, and I have a bad feeling about that.* |
| Lo5 | *I feel unhappy because I am so withdrawn.* |
| Lo6 | I have not been able to make a connection with my fellow students, and this makes me feel lonely. |

*Note: Items printed in italics were later, based on reliability and factor analysis, removed.*
